# Energetics of an rf SQUID Coupled to Two Thermal Reservoirs

**DOI:** 10.1371/journal.pone.0143912

**Published:** 2015-12-07

**Authors:** B. Gardas, J. Łuczka, A. Ptok, J. Dajka

**Affiliations:** 1 University of Silesia, 40-007 Katowice, Poland; Theoretical Division, Los Alamos National Laboratory, Los Alamos, NM 87545, United States of America; 2 University of Silesia, 40-007 Katowice, Poland; Silesian Center for Education and Interdisciplinary Research, University of Silesia, 41-500 Chorzów, Poland; 3 Institute of Nuclear Physics, Polish Academy of Sciences, ul. Radzikowskiego 152, 31-342 Kraków, Poland; Boston College, UNITED STATES

## Abstract

We study energetics of a Josephson tunnel junction connecting a superconducting loop pierced by an external magnetic flux (an rf SQUID) and coupled to two independent thermal reservoirs of different temperature. In the framework of the theory of quantum dissipative systems, we analyze energy currents in stationary states. The stationary energy flow can be periodically modulated by the external magnetic flux exemplifying the rf SQUID as a quantum heat interferometer. We also consider the transient regime and identify three distinct regimes: monotonic decay, damped oscillations and pulse-type behavior of energy currents. The first two regimes can be controlled by the external magnetic flux while the last regime is robust against its variation.

## Introduction

Current tendency toward miniaturization requires a better understanding of phenomena and processes in small space and time domain. In particular, one can notice an upsurge of interest in energy transport phenomena in micro- and nanostructures like molecular junctions, suspended nanotubes, quantum point contacts, nono-wires, phononic metamaterials, etc. [1, 2]. Recent progress and rapid developments in technology will, sooner rather than later, allow to convert research findings into potential applications of functional thermal devices such as quantum heat engines [3], thermal rectifiers [4, 5], thermal transistors [6, 7], controllers of local temperature [8], heat—voltage converters, thermal circuits [9, 10], nano-refrigerators [11] and last but not least hybrid thermal circuits and microelectromechanical machines where energy transfer can be exploited to move microscopic devices.

In this paper we study energy transport in a temperature-biased Josephson junction ring, i.e. a superconducting loop interrupted by a Josephson junction (such a system is also called a rf SQUID). Josephson junctions form a reach family which exhibits interesting both classical and quantum phenomena. They are elements of many low-temperature devices and offer a number of beneficial applications. They are applied in quantum computing devices as single units of information (qubits). Circuits made of Josephson junctions have been turned into artificial atoms which can be manipulated and measured by methods developed in atomic physics. Of special interest are novel heat transport phenomena study of which still is at the initial stadium. A pioneering work in this subject was performed in Ref. [12] and in a series of papers by Guttman *et al*. [13–15]. A more complete and recent literature survey is presented in Refs. [16, 17].

To some extend our studies have been inspired by *coherent caloritronics* and experiments with the dc SQUID [16]. Here, we consider a purely quantum model of the rf SQUID. However, this model is much more general: it is a quantum nonlinear oscillator widely studied in various contexts in nonlinear quantum optics, condensed matter physics, statistical physics and cold atoms physics. In a general context, we study energy transport passing through the nonlinear oscillator which is coupled to two thermal Markovian reservoirs of different temperatures. The applied theoretical approach provides information on the non-equilibrium states of the system, its dynamics, transient and stationary phenomena and influence of the initial state of the system. Therefore this method has the distinct advantage over other methods in use. We examine energy current as it evolves in time from the transient regime to the long-time stationary state. We find several different regimes in the steady-state as well as in the transient time-domain.

The sketch of the paper is as follows. First we briefly present main elements of quantum modelling for the rf SQUID. Next we formulate the corresponding model of Markovian dynamics for an open system weakly coupled to two thermostats of different temperature. Further we present general remarks on energy flow in the system and analyze in detail stationary energy flow as a function of control parameters. In particular, the dependence on the external magnetic flux exhibits periodic behavior and agrees with what was already known in literature and confirmed in experiments [16]. Remaining part of the work is devoted to transient effects: how energy flow depends on time. Finally we provide a summary and conclusions.

## Materials and Methods

### Quantum model of rf SQUID

**Fig 1:**
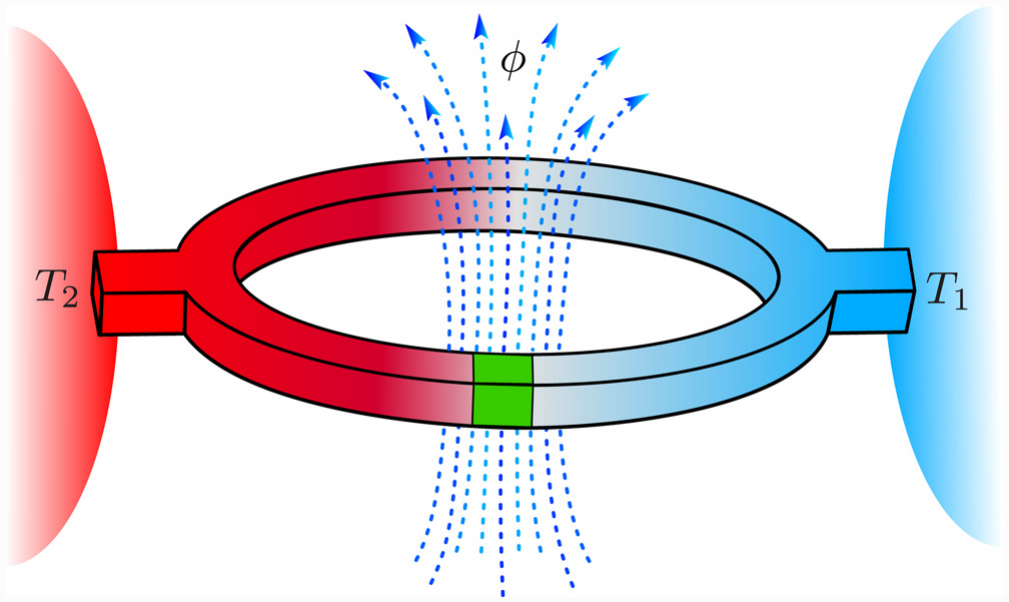
Schematic representation of an rf SQUID in contact with two thermal reservoirs of different temperature *T*_1_ and *T*_2_. The loop is threaded with a magnetic flux *ϕ*.

Now, we present details of the model, see Fig 1. It is a superconducting loop of inductance *L* comprising a single Josephson tunnel junction with critical current *I*_*c*_. The loop is threaded with a magnetic flux *ϕ*. The phase difference *ψ* of the Cooper pair wave function across the junction is related to the magnetic flux *ϕ* via the relation [18]

$$\begin{array}{c} \psi =2\pi (n-\phi /\phi_{0}), \end{array}$$ (1)

where 2*πn* is the phase change per cycle around the loop and in the SI units *ϕ*_0_ = *h*/2*e* is the superconducting flux quantum (the factor of two arises from electron pairing). When the external magnetic field is applied, the total flux consists of two parts, namely,

$$\begin{array}{c} \phi =\phi_{e}+LI, \end{array}$$ (2)

where *ϕ*_*e*_ is the external static flux and *I* is the total current flowing in the loop. We model the system in terms of the Hamiltonian [19]

$$\begin{array}{c} \hat{H}=\frac{Q^{2}}{2C}+\frac{1}{2L}{\left(\phi -\phi_{e}\right)}^{2}-E_{J}\cos\left(2\pi \frac{\phi }{\phi_{0}}\right). \end{array}$$ (3)

This Hamiltonian contains three energy scales entering charging energy *E*_*C*_, inductive energy *E*_*L*_ and Josephson energy *E*_*J*_, respectively. The junction capacitor charge *Q* is canonically conjugated to the flux *ϕ* and the commutation relation for the quantum operators *Q* and *ϕ* assumes the form [*ϕ*, *Q*] = *i*ℏ. The Josephson energy *E*_*J*_ is related to the critical Josephson current *I*_*J*_ via the relation *E*_*J*_ = *ϕ*_0_ *I*_*J*_/2*π*.

We transform Hamiltonian Eq (3) to the form

$$\begin{array}{c} H=U^{†}\hat{H}U=\frac{Q^{2}}{2C}+\frac{\phi^{2}}{2L}-E_{J}\cos\left(2\pi \frac{\phi +\phi_{e}}{\phi_{0}}\right), \end{array}$$ (4)

where the unitary operator *U* = exp(−*iϕ*_*e*_ *Q*/ℏ). Such form of Hamiltonian allows to distill the role of the external flux *ϕ*_*e*_ piercing the SQUID.

In the limit *E*_*J*_ → 0, Eq (4) reduces to the equation of motion for a harmonic oscillator of mass *M* = *C* and frequency $\omega =1/\sqrt{LC}$. By analogy to the harmonic oscillator, we introduce a corresponding Hamiltonian in the form [21]

$$\begin{array}{ccc} H & = & \hbar \omega \left(a^{†}a+\frac{1}{2}\right)\\ & - & E_{J}\cos\left[\frac{2\pi }{\phi_{0}}\sqrt{\frac{\hbar }{2C\omega }}\left(a^{†}+a\right)+2\pi \frac{\phi_{e}}{\phi_{0}}\right], \end{array}$$ (5)

where the annihilation and creation bosonic operators are defined as [21]

$$\begin{array}{c} a=\sqrt{\frac{C\omega }{2\hbar }}\left(\phi -\frac{i}{C\omega }Q\right),\;a^{†}=\sqrt{\frac{C\omega }{2\hbar }}\left(\phi +\frac{i}{C\omega }Q\right). \end{array}$$ (6)

The dimensionless form of the Hamiltonian Eq (5) reads

$$\begin{array}{c} H=\frac{H}{\hbar \omega }=a^{†}a+\frac{1}{2}-E_{J}\cos\left[2\sqrt{E_{C}}\left(a^{†}+a\right)+2\pi \Phi_{e}\right], \end{array}$$ (7)

where the dimensionless parameters

$$\begin{array}{c} E_{J}=\frac{E_{J}}{\hbar \omega },\;E_{C}=\frac{e^{2}/2C}{\hbar \omega },\;\Phi_{e}=\frac{\phi_{e}}{\phi_{0}} \end{array}$$ (8)

have obvious meanings: *ε_J_* is the dimensionless Josephson energy, *ε_C_* is the dimensionless charging energy and *Φ*_*e*_ is the external flux in units of the magnetic flux quantum *ϕ*_0_.

### SQUID in contact with two reservoirs

We now assume that the system under consideration is an open system, i.e. one side of the Josephson junction is kept at temperature *T*_1_ and the other—at *T*_2_, see Fig 1. These temperatures are controlled by two independent thermal reservoirs *1* and *2* of temperature *T*_1_ and *T*_2_, respectively. If temperatures are different, there is heat flow between two reservoirs mediated by the SQUID. The influence of the SQUID on the proces of heat flow is an interesting and difficult problem. In order to describe it, one should model two reservoirs in the framework of microscopic quantum many body systems *a la* Calderia-Legget Hamiltonian [20] and solve a Liouville-von Neumann equation for the total closed system: the SQUID + two reservoirs. However, it is imossible to solve it and therefore some approximations have to be applied. In the paper, we do not analyze the heat flow between two reservoirs but only energy change of the SQUID mediated by two reservoirs. We consider the system weakly interacting with reservoirs *1* and *2*. In the sequel, we adopt the theory of quantum open systems in the weak-coupling regime [26]. This theory is valid under the condition that the relaxation time of heat baths are much shorter than the characteristic time of the system which can be identified with the inverse of the frequency *ω*. In such a case the dynamics is Markovian and governed by a completely positive linear map acting on the reduced density operator *ρ*(*t*) of the system. We follow the idea of Alicki [27] who studied a quantum open system coupled to several reservoirs as a model of a heat engine. In his approach, the reduced dynamics of the system alone is descibed by a master equation [27]

$$\begin{array}{c} \frac{d\rho (t)}{dt}=-i\left[H,\rho \left(t\right)\right]+D_{1}\left[\rho \left(t\right)\right]+D_{2}\left[\rho \left(t\right)\right]. \end{array}$$ (9)

The first term -i[H,ρ(t)] describes the unitary evolution of the density matrix *ρ*(*t*) governed by the dimensionless Hamiltonian H defined in Eq (7) and the dimensionless time *t* = *ωτ*, where *τ* is the dimensional (original) time. The dissipative generators *D*_*i*_ (*i* = 1, 2) describe the influence of thermal reservoirs. In the microscopic formulation, the form of dissipators *D*_*i*_ should be derived from the original physical picture by exploiting e.g. the Davies theory [28]. To apply this theory, the spectral decomposition of the Hamiltonian Eq (7) is needed. However, its explicit form is not known. Therefore we make the next approximation and choose the dissipators in the form well-known in quantum optics [26], namely,

$$\begin{array}{ccc} D_{i}\left(\rho \right) & = & \gamma_{i}\left(N_{i}+1\right)\left(2a\rho a^{†}-a^{†}a\rho -\rho a^{†}a\right)\\ & + & \gamma_{i}N_{i}\left(2a^{†}\rho a-aa^{†}\rho -\rho aa^{†}\right),\;\;\;i=1,2, \end{array}$$ (10)

where *N*_*i*_ is related to temperature *T*_*i*_ and the frequency *ω*_*B*_ of the i-th monochromatic reservoir via the expression

$$\begin{array}{c} N_{i}={\left[\exp\frac{\hbar \omega_{B}}{k_{B}T_{i}}-1\right]}^{-1}. \end{array}$$ (11)

The parameter *γ*_*i*_ is a coupling constant of the system to its *i*-th reservoir.

We are aware that a single mode monochromatic reservoirs are to crude for analysis of the heat flow between two reservoirs. However, for the energy change of the system alone it can be admissible to give insight into oscillation and relaxation phenomena. Moreover, a similar approach has been used in Ref. [21] to demonstrate the manner in which decoherence affects the quantum states of the loop. Lately [22], the same dissipators have been applied with success for analysis of the quantum dynamics of two electromagnetic oscillators coupled in series to a voltage-biased Josephson junction. The similar scheme has been proposed for a quantum thermal machine made by atoms interacting with a single nonequilibrium electromagnetic field [23]. The next system is a realistic device to reversibly extract work in a battery of finite energy: a hybrid optomechanical system [24]. Such devices consist of an optically active two-level quantum system interacting strongly with a nano-mechanical oscillator that provides and stores mechanical work, playing the role of the battery. Again, the authors of Ref. [24] exploite the same form of dissipators. Finally, we would like to mention the paper [25] in which time-resolved statistics of nonclassical light in Josephson photonics has been studied in the framework of the Lindblad master equation. The above examples show that the dissipators Eq (10) are universal and in many cases can correctly describe the influence of environment.

### Energy flow

The change of averaged energy E(t)=Tr[Hρ(t)] of the SQUID is determined by the master Eq (9) and reads [26]

$$\begin{array}{c} \frac{dE(t)}{dt}=J\left(t\right)=J_{1}\left(t\right)+J_{2}\left(t\right). \end{array}$$ (12)

The energy current *J*_1_(*t*) is related to the change of energy of the SQUID caused by the coupling to the reservoir *1* and is given by the relation

$$\begin{array}{c} J_{1}\left(t\right)=\text{Tr}\left\{HD_{1}\left[\rho \left(t\right)\right]\right\}. \end{array}$$ (13)

In turn, the energy current *J*_2_(*t*) is related to the change of energy due to the coupling of the SQUID to the reservoir *2* and has the form

$$\begin{array}{c} J_{2}\left(t\right)=\text{Tr}\left\{HD_{2}\left[\rho \left(t\right)\right]\right\}. \end{array}$$ (14)

The dimensionless Hamiltonian Eq (7) is a periodic function of the magnetic flux *Φ*_*e*_ with period *L* = 1, i.e. $H\left(\Phi_{e}\right)=H\left(\Phi_{e}+1\right)$. In consequence, averaged values of observables are also periodic. In particular, the energy currents are periodic with respect to *Φ*_*e*_.

In the thermalization process, no work is performed and heat flows between reservoirs *1* and *2* through the SQUID. In the stationary state, when *t* → ∞, the statistical operator *ρ*(*t*)→*ρ*. In consequence *dE*(*t*)/*dt* = 0 and from Eq (12) it follows that the total energy current *J*(*t*)→*J* = *J*_1_ + *J*_2_ = 0. Hereafter, we will consider the case *T*_2_ > *T*_1_. Therefore the stationary energy current *J*_2_ > 0 (heat is flowing from the reservoir *2* to the SQUID) and *J*_1_ < 0 (heat is flowing from the SQUID to the reservoir *1*).

We have solved the master Eq (9) numerically by truncation the infinite—dimensional Hilbert space at *dim*_*max*_ = 100. Next, we have solved a remaining system of differential equations using a variable—order and variable—step Adams method [29]. We have tested this procedure and ascertained that for considered parameters it allows to obtain converged and stable results. Parts of our so obtained results are presented next.

## Results and Discussion

### Stationary regime

**Fig 2:**
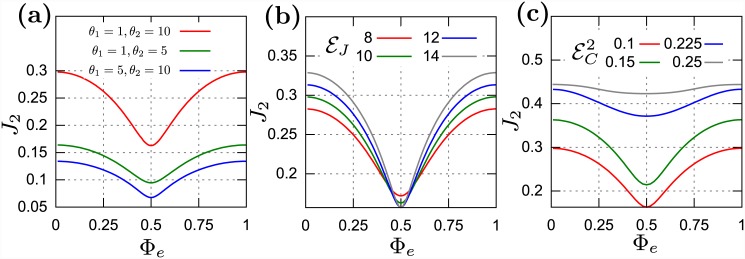
The stationary energy current *J*_2_ as a function of the external magnetic flux Φ_*e*_. Panel (a) for various temperatures of reservoirs: ({*θ*_1_, *θ*_2_} = {1, 10}, {1, 5}, {5, 10}. Panel (b) for $E_{C}^{2}$ = 0.1 and various Josephson energy: *ε_J_* = 8, 10, 12, 14. Panel (c) for *ε_J_* = 14 and various charging energy $E_{C}^{2}$ = 0.1, 0.15, 0.225, 0.25.

**Fig 3:**
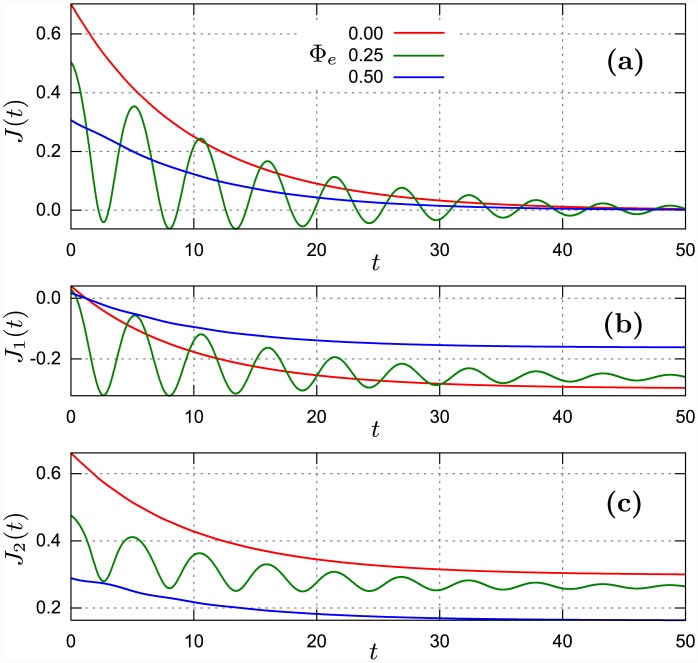
Short-time regime of (a) the total energy current *J*(*t*), (b) the energy current *J*_1_(*t*) induced only by the reservoir of temperature *T*_1_ and (c) the energy current *J*_2_(*t*) induced by the reservoir of temperature *T*_2_ for selected values of the rescaled external magnetic flux Φ_*e*_. The reservoir rescaled temperatures are *θ*_1_ = *k*_*B*_ *T*_1_/ℏ*ω*_*B*_ = 1, *θ*_2_ = *k*_*B*_ *T*_2_/ℏ*ω*_*B*_ = 10. Remaining parameters are fixed as: *ε_J_* = 10, $\;E_{C}^{2}$ = 0.1 and *γ*_1_ = *γ*_2_ = 0.05. The dimensionless time *t* is rescaled by the oscillator frequency *ω*.

Let us study generic properties of the long-time stationary energy change of the SQUID as a function of selected parameters of the model. In Figs 2 and 3 one can monitor the energy current approach to the stationary state. Because the total stationary energy current *J* = *J*_1_ + *J*_2_ = 0, we analyze only the current *J*_2_ > 0 (under the assumption *T*_2_ > *T*_1_). It is a periodic function of the magnetic flux, *J*_2_(*Φ*_*e*_) = *J*_2_(*Φ*_*e*_ + 1). There are regimes where *Φ*_*e*_ is a relevant control parameter. In Fig 2 we display the dependence of the stationary current *J*_2_ on *Φ*_*e*_. Let us notice that the energy current can be reduced by an external magnetic field by more that 50%. This opens a door for applications as a energy controller in small devices: by continuous changing of the external magnetic field, one can heat up or cool down the system. But this is not always the case because there are regimes where *J*_2_ weakly depends on *Φ*_*e*_ and then *Φ*_*e*_ is an irrelevant control parameter, i.e. the energy current depends so weakly on *Φ*_*e*_ that it would be difficult to detect this behavior.

In Fig 2 we also show how the stationary energy current depends on the scaled Josephson energy *ε_J_* and the scaled charging energy *ε_C_*. The increase of *ε_J_* leads to a greater amplitude of oscillations of *J*_2_. It seems to be related to the form of the Hamiltonian Eq (7) in which *ε_J_* plays a role of amplitude in the cosine part. The greater amplitude of oscillations in *J*_2_ allows for a greater reduction of energy current by *Φ*_*e*_. In turn, the change of the charging energy *ε_C_* is not so evident. From panel (c) of Fig 2 one can notice that the variation of *ε_C_* can increase the amplitude of the *J*_2_-oscillations in a narrow interval of *ε_C_*. Outside this interval, monotonic changes of *ε_C_* result in flattening of modulation of *J*_2_ and the interference effects are masked and cannot be detectable.

### Time-dependent regime

**Fig 4:**
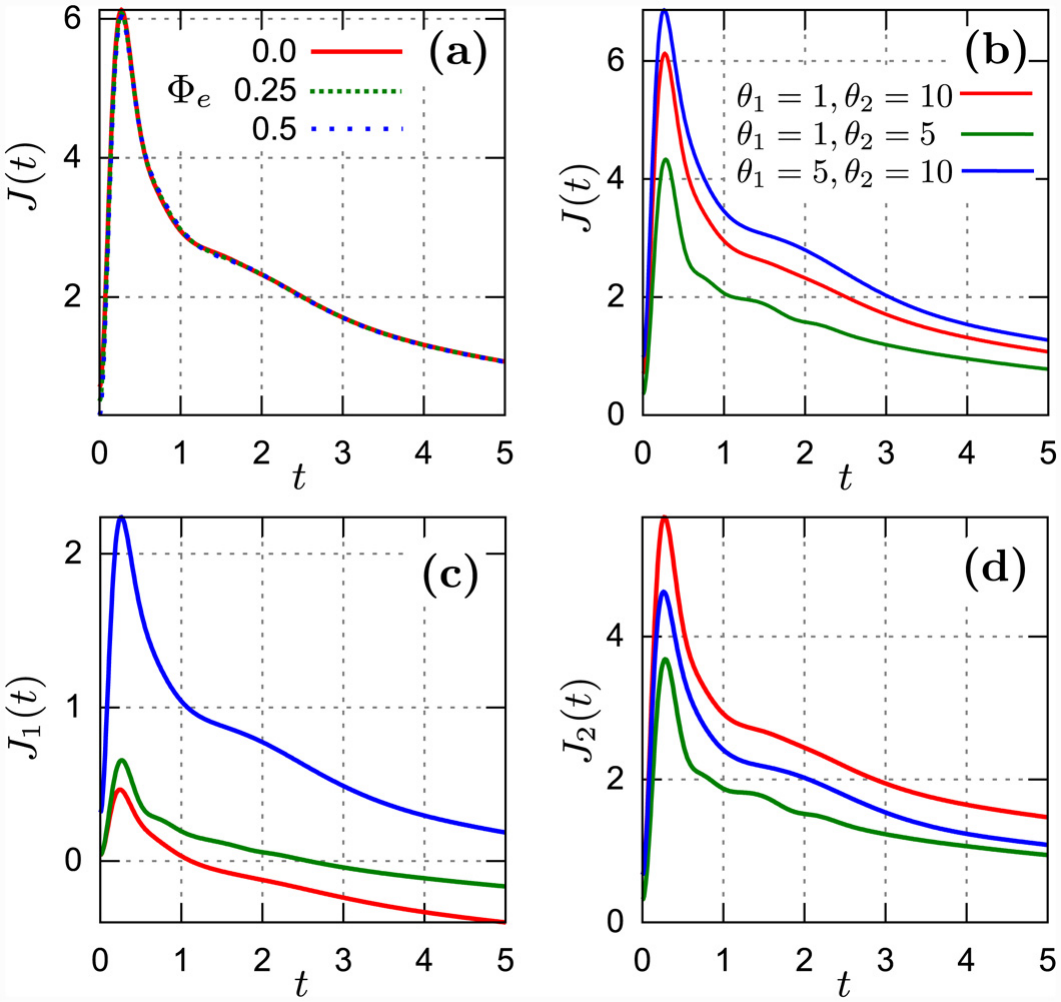
Pulse regime. (a) The total energy current *J*(*t*) = *J*_1_(*t*) + *J*_2_(*t*) as a function of time for three values of the external magnetic flux Φ_*e*_ = 0, 0.25, 0.5. The reservoir rescaled temperatures are *θ*_1_ = 1, *θ*_2_ = 10. (b) *J*(*t*) is shown for three sets of reservoirs temperatures {*θ*_1_, *θ*_2_} = {1, 10}, {1, 5}, {5, 10}. (c) *J*_1_(*t*) and (d) *J*_2_(*t*) for the same set of temperatures as in panel (b). In panels (b)-(d) the flux Φ_*e*_ = 0. Remaining parameters are fixed as: *ε_J_* = 4, $E_{C}^{2}$ = 1.5 and *γ*_1_ = *γ*_2_ = 0.05. The dimensionless time *t* is rescaled by the oscillator frequency *ω*.

In this section we analyze an approach to the stationary regime. To this aim, we have solved numerically the master Eq (9) with an initial condition *ρ*(0). As an initial state of the system Eq (7), we consider the vacuum state *ρ*(0) = |0〉〈0|. Therefore both the total current *J*(*t*) and partial currents *J*_1_(*t*) and *J*_2_(*t*) are positive for very short time *t* > 0. It means that initially energy is pumped from both reservoirs to the SQUID. Next, one can observe several distinct evolution of the energy currents: (A) a monotonically decreasing function of time (as in the case *Φ*_*e*_ = 0 in Fig 3) or non-monotonic approach to the stationary flow. In turn, the non-monotonic time evolution can exhibit: (B) damped oscillations (the case *Φ*_*e*_ = 0.25 in Fig 3) or: (C) pulse-type evolution (Fig 4). In some regimes, the external magnetic flux *Φ*_*e*_ has no qualitative but only small quantitative influence on the amplitude of energy currents (as in Fig 3). Most interesting is the regime (B). By varying the magnetic field one can cause drastic changes in energy flow, as it is visualized in Fig 3. For three values of the external magnetic flux *Φ*_*e*_ = 0, 1/4 and 1/2, the transition form monotonic to oscillatory and next again to monotonic flow is detected. If next one increases *Φ*_*e*_ from the value 1/2 through 3/4 to 1, the reversed behavior is observed. Because *T*_2_ > *T*_1_ the energy flows from the reservoir *2* through the SQUID to the reservoir *1*. In a generic case, the averaged energy *E*(*t*) of the SQUID should grow in time to a constant stationary value *E*. In the case presented in Fig 3, the total current *J*(*t*) takes both positive and negative values. It means that there are time intervals in which the energy *E*(*t*) increases and there are time intervals in which the energy *E*(*t*) decreases. The phenomenon is quasi-periodic in time. It is an evident signature of the quantum interference effects in energy transport through the SQUID.

In Fig 4 we illustrate the pulse-type regime (C): at short time the energy current grows rapidly and after reaching its maximal value at some characteristic time it starts to decrease until a steady state is reached. In this case we find that the energy flow is robust against variation of the magnetic flux *Φ*_*e*_. The magnitude of the pulse depends rather on both temperatures *T*_1_ and *T*_2_ and not on the temperature difference *T*_1_ − *T*_2_. Let us notice that in all three figures for *J*(*t*), *J*_1_(*t*) and *J*_2_(*t*), the sequence of colors is different. E.g. the maximal magnitude of the pulse for *J*(*t*) and *J*_1_(*t*) is noticed for the set {*T*_1_ = 5, *T*_2_ = 10}; for the current *J*_2_(*t*)—for the set {*T*_1_ = 1, *T*_2_ = 10}. In turn, the minimal magnitude of the pulse for *J*(*t*) and *J*_2_(*t*) is observed for the set {*T*_1_ = 1, *T*_2_ = 5} while for *J*_1_(*t*) it is for the set {*T*_1_ = 1, *T*_2_ = 10}.

Ending this section, we have to discuss the question of applicability of the Markovian master equation to time-dependent regimes. Starting from a giving initial state, for very short time its evolution is non-Markovian. It can be approximated by a Markovian process for time longer than the relaxation time of the system. We extrapolate results for short time but we are not able to prove that it is compatible with the description considerd in the paper. Only comparison with results obtained from the exact reduced dynamics could answer this question. However, the exact reduced dynamics is not known and the short-time regimes should be treated with limited trust.

### Summary

We studied the energy flow in a microscopic, fully quantum-mechanical dissipative model of an rf SQUID coupled to two heat baths. The results have implications beyond this system because the Hamiltonian Eq (3) describes several other quantum systems. In the stationary regime, the energy flow can be periodically modulated by *Φ*_*e*_ exemplifying the rf SQUID as a quantum energy interferometer. We have found three different time-dependent regimes of the energy current: monotonic decay, damped oscillations and pulse-type behavior. The first two regimes are sensitive to change of *Φ*_*e*_. This feature allows to manipulate and control the energy flow by the external magnetic field. The total current can take both positive and negative values and in consequence there are time intervals in which the energy of the SQUID increases and there are time intervals in which the energy decreases. The phenomenon is quasi-periodic in time. It is an evident signature of the quantum interference effects in energy transport through the SQUID. The pulse regime is robust against variation of *Φ*_*e*_. The magnitude and to some extent also the position of the pulse can be controlled by parameters of the model. Recent progress in novel experimental techniques makes the verification of our findings quite realistic. Nowadays, the system can be realized [16] and researchers can observe heat processes over time scales ranging between a few picoseconds and a few nanoseconds [30].
